# Psychometric Evaluation of the HIV Stigma Scale in a Swedish Context

**DOI:** 10.1371/journal.pone.0114867

**Published:** 2014-12-18

**Authors:** Maria H. Lindberg, Lena Wettergren, Maria Wiklander, Veronica Svedhem-Johansson, Lars E. Eriksson

**Affiliations:** 1 Department of Neurobiology, Care Sciences and Society, Karolinska Institutet, Huddinge, Sweden; 2 Department of Clinical Sciences, Danderyd Hospital, Karolinska Institutet, Stockholm, Sweden; 3 Department of Infectious Diseases, Karolinska University Hospital, Huddinge, Sweden; 4 Unit of Infectious Diseases, Department of Medicine Huddinge, Karolinska Institutet, Stockholm, Sweden; 5 School of Health Sciences, City University London, London, United Kingdom; University of Perugia, Italy

## Abstract

**Background:**

HIV-related stigma has negative consequences for infected people's lives and is a barrier to HIV prevention. Therefore valid and reliable instruments to measure stigma are needed to enable mapping of HIV stigma. This study aimed to evaluate the psychometric properties of the HIV stigma scale in a Swedish context with regard to construct validity, data quality, and reliability.

**Methods:**

The HIV stigma scale, developed by Berger, Ferrans, and Lashley (2001), was distributed to a cross-sectional sample of people living with HIV in Sweden (n = 194). The psychometric evaluation included exploratory factor analysis together with an analysis of the distribution of scores, convergent validity by correlations between the HIV stigma scale and measures of emotional well-being, and an analysis of missing items and floor and ceiling effects. Reliability was assessed using Cronbach's α.

**Results:**

The exploratory factor analysis suggested a four-factor solution, similar to the original scale, with the dimensions *personalised stigma*, *disclosure concerns*, *negative self-image*, and *concerns with public attitudes*. One item had unacceptably low loadings and was excluded. Correlations between stigma dimensions and emotional well-being were all in the expected direction and ranged between −0.494 and −0.210. The instrument generated data of acceptable quality except for participants who had not disclosed their HIV status to anybody. In line with the original scale, all subscales demonstrated acceptable internal consistency with Cronbach's α 0.87–0.96.

**Conclusion:**

A 39-item version of the HIV stigma scale used in a Swedish context showed satisfactory construct validity and reliability. Response alternatives are suggested to be slightly revised for items assuming the disclosure of diagnosis to another person. We recommend that people that have not disclosed should skip all questions belonging to the dimension personalised stigma. Our analysis confirmed construct validity of the instrument even without this dimension.

## Background

About 35.3 million people were living with HIV worldwide in 2012, according to UNAIDS [1]. Currently, there are 6,469 known people living with HIV in Sweden (63% male, 37% female) [2]. Since the introduction of combined antiretroviral treatment (cART), HIV has changed from being a potentially deadly disease to a chronic disease in countries where cART is generally available to those in need. In Sweden, cART is available free of cost, and 93% of the population diagnosed with HIV is undergoing treatment. Patients without treatment often have a CD4^+^ T-cell count above 500×10^6^ cells/ml and are therefore currently not considered in need of treatment [2].

HIV-related stigma has been a barrier to HIV prevention since the beginning of the pandemic and has been shown to have negative effects on care and treatment, i.e. lower rates of HIV testing and lower adherence to medication [3], [4], [5]. Stigma, as defined by Goffman, appears when an attribute becomes deeply discrediting within certain relations and contexts [6]. HIV stigma is considered a social phenomenon, grounded on the labelling and stereotyping of people living with HIV, leading to loss of status and discrimination [7]. HIV stigma experienced by people living with HIV can be enacted, anticipated or internalised. Enacted stigma involves experiences of discrimination, stereotyping and or prejudice from others due to one's HIV infection. Anticipated stigma includes expectations of enacted stigma. Internalised stigma refers to a situation when stereotyping and or prejudice involving negative feelings and beliefs about people living with HIV have been internalised by people living with HIV [8].

Valid and reliable instruments for measuring stigma are needed to be able to map HIV stigma in affected populations as a base for the development of interventions against stigma and to evaluate the effects of stigma-reducing interventions [9]. According to Earnshaw and Chaudoir [8] it is important that such an instrument can differentiate between stigma mechanisms to identify the mechanism(s) that should be targeted in a potential intervention. Several instruments are designed to measure HIV-related stigma (see e.g. [8] for an extensive review), for example the HIV stigma scale by Sowell et al [10], the Internalized stigma scale by Sayles et al [11], the Measures of stigma and social impact of disease by Fife and Wright [12], the Enacted, vicarious, felt normative and internalized HIV stigma scales by Steward et al [13], the Stigma mechanisms of the HIV stigma framework by Earnshaw et al [14] and the HIV stigma scale by Berger et al [15]. However, only the HIV sigma scale designed by Berger et al [15] both differentiates between the three stigma mechanisms proposed by Earnshaw and Chaudoir [8] in one single instrument and produces an overall HIV stigma score in addition to the different stigma dimension scores. It has been used to measure stigma in various populations including African American women [16], men who have sex with men (MSM) [17] and adults 50 years and older [18] in the US and in men and women in Kenya and Puerto Rico [19]. Short versions of the scale have been developed in English and Swedish to measure HIV-related stigma among children and adolescents [20], [21]. As no instruments for the measurement of HIV-related stigma among adults are available for a Swedish context, this study was set out to evaluate the psychometric properties of the HIV stigma scale in a Swedish context with regard to construct validity, data quality and reliability.

## Method

### The HIV Stigma Scale

The HIV stigma scale consists of 40 items that form four subscales and an overall scale [15]. The development of the original HIV stigma scale was based on an extensive literature review regarding HIV-related stigma and psychosocial aspects of living with HIV as well as the involvement of experts and HIV-related organisations across the United States. Exploratory factor analyses of the original English version resulted in four factors representing four dimensions of stigma: (1) *personalised stigma*, (2) *disclosure concerns*, (3) *negative self-image* and (4) *concerns with public attitudes*, each composing a subscale of the instrument. The *personalised stigma* dimension is proposed to represent the enacted stigma mechanism, *concerns with public attitudes* and *disclosure concerns* are proposed to represent the anticipated stigma mechanism and *negative self-image* is proposed to represent the internalised stigma mechanism [8]. The 40 items are statements that a person living with HIV can agree or disagree with on four-point Likert-type response alternatives (completely disagree, disagree, agree and completely agree). Seventeen of the items in the instrument are statements that include an assumption of the disclosure of one's HIV status, at least to some extent; in a written instruction prior to this section, the participant is asked to imagine the situation if no one else knows that he or she has HIV. Subscale scores are calculated by summing the scores for the items belonging to each subscale, and an overall stigma score is calculated by summing the ratings for all 40 items. The instrument was originally tested in a sample of 318 persons (81% men) living with HIV in the US and showed satisfactory internal consistency for the subscales and overall scores with coefficient α ranging from 0.90 to 0.93. The test-retest reliability ranged from 0.89 to 0.92 with 2–3 weeks between tests. HIV-related stigma is hypothesised to be negatively related to self-esteem and positively related to depression, and in line with these assumptions, moderate to strong correlations between the HIV stigma scale and measures of self-esteem, depression and aspects of social support and conflict have been found, supporting the construct validity of the instrument [15].

#### Translation of the HIV Stigma Scale to Swedish

The 40 items were translated independently from English into Swedish by three members of the research group, all well experienced within the area of infectious diseases. The three translated versions were compared and merged into one Swedish version that was reviewed by a bilingual consultant (Swedish–English). This was followed by minor changes before the items were translated back into English by a professional translator and compared to the original scale. Additional small changes were conducted to ensure that the Swedish version did not differ from the original instrument.

#### Feasibility of the Swedish HIV Stigma Scale

The feasibility of the items was assessed through think-aloud interviews with a purposeful sample of people living with HIV (7 men, 2 women; 3 born in Sweden, 6 born in other countries) who completed the Swedish version of the HIV stigma scale whilst sharing their thoughts aloud [22], [23]. The analysis showed that the participants overall found the items relevant and comprehensive.

### Sample and Procedure

Data were collected from March through September 2013. Participants were recruited from the Department of Infectious Diseases at the Karolinska University Hospital in Stockholm, Sweden; the sample of patients listed at the clinic was judged representative regarding gender distribution and immigration status for people living with HIV in Sweden. The inclusion criteria were: (1) diagnosis with HIV and (2) 18 years of age and older. Patients who were newly diagnosed (<6 months) or had their first appointment at the clinic were excluded. A member of the research team approached eligible participants when they came to the clinic for scheduled appointments, and patients who accepted participation responded to the instrument either at the clinic or at home. Participants with insufficient knowledge in Swedish or English were offered the opportunity to fill out the questionnaire with a professional translator or with a member of the research team. The assistance from the research team was individualized and included explanations of the statements and response alternatives. At the end of the inclusion period, a shortage of men was noticed, so efforts were made to reach this group through purposive recruitment.

### Additional Instruments

All participants were in addition to the HIV stigma scale asked to complete the Swedish Health-related Quality of Life Survey (Swed-Qual) [24]. Swed-Qual was derived from the Medical Outcome Study, MOS, consisting of 63 items and forming 13 scales covering physical, mental, social and general health. Swed-Qual has previously been used to measure quality of life among people living with HIV [25], [26]. Two of the eleven multi-item scales from Swed-Qual, emotional well-being, negative effect and emotional well-being, positive effect were hypothesised to be associated with stigma mechanisms and used to investigate the construct validity of the HIV stigma scale. The emotional well-being, negative effect and emotional well-being, positive effect consists of six statements respectively (e.g. *I have felt down* and *I have felt harmony*). Each statement is rated on a four point Likert scale ranging from “completely agree” to “completely disagree”. The answers are transformed to a 0–100 scale where 0 indicates worst possible and 100 best possible health-related quality of life; the two scales are presented as mean scores from the answers of the items belonging to the respective scale.

### Data Analysis

Statistical analyses with the exception of parallel analysis were conducted in IBM SPSS Statistics 22. Randomised eigenvalues for parallel analysis were derived using the package nFactors in R Statistics [27], [28].

#### Construct Validity

To explore the latent structure of the data set, an exploratory factor analysis was performed. The adequacy of the data for factor analysis was investigated with the Kaiser-Meyer-Olkin measure of sampling adequacy (KMO) and Bartlett's test of sphericity [29]. Alpha factoring with oblimin rotation was used as the extraction method to simulate the analysis performed by Berger et al. [15]. The number of factors extracted was determined through parallel analysis [30] and a screeplot [29]. The pattern matrix was analysed regarding loadings; only items with loadings of >0.32 [29] were included in the final version. An item with two or more loadings >0.32 was considered a cross-loading item [29] and assigned to the single factor with the highest loading. In the same way, an additional factor analysis was performed without the 16 items that loaded on the dimension personalised stigma to secure construct validity without this dimension.

The distribution of scores within the subscales was evaluated through means and standard deviations on the subscale and item levels. Item means and standard deviations were expected to be roughly equivalent within the subscale to justify the summation of item scores into subscale scores [31]. Corrected item-total correlation coefficients were examined and expected to exceed 0.4.

Convergent validity was assessed by the Spearman's rank correlation coefficient between the HIV stigma scale and selected subscales from Swed-Qual: emotional well-being, negative effect and emotional well-being, positive effect. In both Swed-Qual subscales, lower scores reflect worse emotional well-being; it was hypothesised that the selected Swed-Qual scales would have moderate correlations with the stigma subscales. Correlation coefficients of 0.10–0.29, 0.30–0.49 and 0.49 and above were interpreted as small, moderate and large, respectively [32].

#### Data Quality

Data quality was evaluated through analysis of missing values for each item. Items that more than 5% of the participants had not answered, i.e. missing values, were further examined to see whether there was reason to believe that the item had been misunderstood or whether there were other explanations for the missing values [33].

The four-point Likert scale of the HIV stigma scale was evaluated through analysis of whether all response alternatives, 1–4, were used for all items. Floor and ceiling effects were calculated and considered acceptable if they did not exceed 15% [34].

#### Reliability

Cronbach's α was calculated for the subscales and for the overall scale to investigate the internal consistency of the scale and considered acceptable if it exceeded 0.7 [29].

### Ethical considerations

The study was approved by the Regional Ethical Review Board of Stockholm [Regionala etikprövningsnämnden i Stockholm], FE 289, SE-171 77 Stockholm, Sweden (record no 2013/335-32) and has been performed in accordance with the ethical standards laid down in the 1964 Declaration of Helsinki and its later amendments. Written informed consent was collected from all participants. Oral and written information about the study was given in Swedish or English to all potential participants. The information included the aims of the study, voluntariness and the possibility to withdraw at any point without any effects on current or future care, the fact that that answers would be treated with confidentiality and that data would be presented only on a group level, with individuals kept anonymous. Participants who could not read Swedish or English received the written information read out loud by a member of the research team or a professional translator.

## Results

One hundred and ninety-four people living with HIV agreed to participate in the study (85 women and 109 men in the ages 19–83 years; mean 48.8, SD 11.7, response rate 53%). Further socio-demographic data of the sample is shown in Table 1. One hundred and sixty-seven of the participants completed the Swedish version and 27 completed the English version of the questionnaire. Eight participants completed the questionnaire with assistance from a professional translator and 34 received assistance from the research team. The χ^2^ test showed that the sample was representative for people living with HIV in Sweden regarding gender and origin [2]. A significant difference was found regarding path of transmission, where an overrepresentation of heterosexual transmission was seen in the sample (60% vs. expected 51%, χ^2^ 8.02, df 3, *p*<0.05).

**Table 1:** Demographic data of the participants (n = 194); path of transmission and origin, by gender.

	Female (n = 85)	Male (n = 109)
**Origin**		
Western and Central Europe	26	78
Eastern Europe, Africa, Asia, Latin America	59	31
**Path of transmission**		
Heterosexual	70	47
Men who have sex with men	NA	53
Intravenous drug use	8	5
Other	7	4

NA Not Applicable.

### Construct Validity

The answers from the participants who responded to all items in the HIV stigma scale (n = 132) were used for the exploratory factor analysis. The dataset proved suitable for exploratory factor analysis with a KMO of 0.910 and statistical significance for Bartlett's test of sphericity (*p*<0.001). Parallel analysis and the screeplot indicated a four-factor solution, with all items loading on the same factor as in the original HIV stigma scale and five items cross-loading (Table 2). The four factors accounted for 62.2% of the total variance. One item, number 11 (*It is easier to avoid new friendships than to worry about telling someone that I have HIV*), had no loadings >0.32 (Table 2) and was not included in further analyses. Each of the remaining 39 items was assigned to a single factor according to their highest loading, as presented in Table 2.

**Table 2:** Factor loadings for all 40 items in the HIV stigma scale.^a^

Items	Component				
	1	2	3	4	Factor assignment^b^
39. People seem afraid of me once they learn I have HIV	0.866				1 (1, 4, 3)
29. People I care about stopped calling after learning I have HIV	0.864				1 (1)
38. People who know I have HIV tend to ignore my good points	0.833				1 (1, 3, 4)
28. Some people avoid touching me once they know I have HIV	0.824				1 (1, 4)
35. I have stopped socialising with some people because of their reactions to my having HIV	0.776				1 (1)
36. I have lost friends by telling them I have HIV	0.770				1 (1)
33. People have physically backed away from me when they learn I have HIV	0.726				1 (1, 4)
24. I have been hurt by how people reacted to learning I have HIV	0.721				1 (1)
32. People don't want me around their children once they know I have HIV	0.714				1 (1, 4)
30. People have told me that getting HIV is what I deserve for how I lived my life	0.700				1 (1, 4)
27. As a rule, telling others that I have HIV has been a mistake	0.677	0.344			1 (1, 4, 3)
31. Some people close to me are afraid others will reject them if it becomes known that I have HIV	0.671				1 (1)
26. I regret having told some people that I have HIV	0.642	0.327			1 (1)
34. Some people act as though it's my fault I have HIV	0.638				1 (1, 4)
40. When people learn you have HIV, they look for flaws in your character	0.627				1 (4, 1)
18. Some people who know I have HIV have grown more distant	0.604				1 (1)
6. I work hard to keep my HIV a secret		0.751			2 (2, 3)
17. I am very careful who I tell that I have HIV		0.746			2 (2)
1. In many areas of my life, no one knows that I have HIV		0.696			2 (2)
21. I never feel the need to hide the fact that I have HIV (R)		0.575	−0.411		2 (2)
4. Telling someone I have HIV is risky		0.614			2 (2, 4)
25. I worry that people who know I have HIV will tell others	0.362	0.542			2 (2)
22. I worry that people may judge me when they learn I have HIV		0.493			2 (2, 4)
37. I have told people close to me to keep the fact that I have HIV a secret		0.402			2 (2)
15. Having HIV makes me feel that I'm a bad person			−0.737		3 (3)
7. I feel I am not as good a person as others because I have HIV			−0.698		3 (3)
3. People's attitudes about HIV make me feel worse about myself			−0.665		3 (3)
8. I never feel ashamed of having HIV (R)			−0.654		3 (3)
12. Having HIV makes me feel unclean			−0.570		3 (3)
2. I feel guilty because I have HIV			−0.532		3 (3)
23. Having HIV in my body is disgusting to me			−0.530		3 (3)
13 Since learning I have HIV, I feel set apart and isolated from the rest of the world	0.357		−0.484		3 (1, 3, 4)
20. Most people are uncomfortable around someone with HIV				−0.769	4 (4)
9. People with HIV are treated like outcasts				−0.639	4 (4)
14. Most people think that a person with HIV is disgusting				−0.613	4 (4)
10. Most people believe that a person who has HIV is dirty				−0.599	4 (4)
16. Most people with HIV are rejected when others find out				−0.598	4 (4, 1)
5. People with HIV lose their jobs when their employers find out				−0.477	4 (4)
19. Since learning I have HIV, I worry about people discriminating against me				−0.389	4 (4, 2)
11. It is easier to avoid new friendships than worry about telling someone that I have HIV					Excluded (3, 2, 4)

a Factor loadings based on alpha factoring with oblimin rotation. Pattern matrix. Factor loadings <0.32 not shown.

b in the Swedish version (factor assignment in original English version).

(R) indicates that the item is reversely scored.

Item means within subscales were roughly equivalent, and the standard deviations were close to one (Table 3). However, the item means differed between subscales, where personalised stigma and negative self-image had the lowest mean scores of 2.16 and 2.17, respectively, and disclosure concerns had the highest mean score of 3.07. Corrected item-total correlations coefficients exceeded 0.4 for all items. Descriptive statistics for the four subscales are shown in Table 4.

**Table 3:** Descriptive statistics for all 39 items in the Swedish version of the HIV stigma scale.

Item	N	Missing values, n (%)	Mean score	SD
***Personalised stigma***				
18. Some people who know I have HIV have grown more distant	183	11 (5.6%)	2.27	1.07
24. I have been hurt by how people reacted to learning I have HIV	183	11 (5.6%)	2.51	1.10
26. I regret having told some people that I have HIV	185	9 (4.6%)	2.56	1.07
27. As a rule, telling others that I have HIV has been a mistake	183	11 (5.6%)	2.22	1.08
28. Some people avoid touching me once they know I have HIV	184	10 (5.2%)	2.04	1.02
29. People I care about stopped calling after learning I have HIV	184	10 (5.2%)	2.00	1.06
30. People have told me that getting HIV is what I deserve for how I lived my life	185	9 (4.6%)	1.78	1.01
31. Some people close to me are afraid others will reject them if it becomes known that I have HIV	182	12 (6.2%)	2.12	1.02
32. People don't want me around their children once they know I have HIV	183	11 (5.6%)	2.05	1.04
33. People have physically backed away from me when they learn I have HIV	179	15 (7.7%)	2.17	0.99
34. Some people act as though it's my fault I have HIV	184	10 (5.2%)	2.23	1.09
35. I have stopped socialising with some people because of their reactions to my having HIV	183	11 (5.6%)	2.21	1.11
36. I have lost friends by telling them I have HIV	180	14 (7.2%)	2.07	1.10
38. People who know I have HIV tend to ignore my good points	182	12 (6.2%)	1.97	1.00
39. People seem afraid of me once they learn I have HIV	179	15 (7.7%)	2.17	1.03
40. When people learn you have HIV, they look for flaws in your character	179	15 (7.7%)	2.23	1.01
***Disclosure concerns***				
1. In many areas of my life, no one knows that I have HIV	190	4 (2.1%)	3.19	1.05
4. Telling someone I have HIV is risky	189	5 (2.6%)	3.04	1.04
6. I work hard to keep my HIV a secret	191	3 (1.5%)	2.98	1.10
17. I am very careful who I tell that I have HIV	190	4 (2.1%)	3.39	0.91
21. I never feel the need to hide the fact that I have HIV (R)	188	6 (3.1%)	3.15	1.05
22. I worry that people may judge me when they learn I have HIV	190	4 (2.1%)	3.00	1.03
25. I worry that people who know I have HIV will tell others	187	7 (3.6%)	2.56	1.12
37. I have told people close to me to keep the fact that I have HIV a secret	181	13 (6.7%)	2.80	1.14
***Negative self-image***				
2. I feel guilty because I have HIV	191	3 (2.2%)	2.28	1.16
3. People's attitudes about HIV make me feel worse about myself	188	6 (3.1%)	2.28	1.10
7. I feel I am not as good a person as others because I have HIV	190	4 (2.1%)	1.99	1.10
8. I never feel ashamed of having HIV (R)	192	2 (1.0%)	2.65	1.14
12. Having HIV makes me feel unclean	189	5 (2.6%)	2.13	1.09
13 Since learning I have HIV, I feel set apart and isolated from the rest of the world	189	5 (2.6%)	2.08	1.02
15. Having HIV makes me feel that I'm a bad person	189	5 (2.6%)	1.86	0.96
23. Having HIV in my body is disgusting to me	189	5 (2.6%)	2.14	1.07
***Concerns with public attitudes***				
5. People with HIV lose their jobs when their employers find out	173	21 (10.8%)	2.38	1.03
9. People with HIV are treated like outcasts	185	9 (4.6%)	2.72	0.89
10. Most people believe that a person who has HIV is dirty	186	8 (4.1%)	2.58	1.00
14. Most people think that a person with HIV is disgusting	185	9 (4.6%)	2.59	0.97
16. Most people with HIV are rejected when others find out	182	12 (6.2%)	2.60	0.88
19. Since learning I have HIV, I worry about people discriminating against me	190	4 (2.1%)	2.75	1.06
20. Most people are uncomfortable around someone with HIV	183	11 (5.7%)	2.78	0.93

(R) indicates that the item is reversely scored.

**Table 4:** Descriptive statistics for the subscales of the HIV stigma scale.

Dimension (n items)	Possible range	Complete answers, n	Mean (SD)	Floor/ceiling effect, %	Reliability, α
Personalised stigma (16)	16–64	155	34.2 (12.8)	4.6/1.5	0.958
Disclosure concerns (8)	8–32	169	24.6 (6.0)	0.5/9.3	0.871
Negative self-image (8)	8–32	180	17.5 (6.4)	7.2/1.5	0.883
Concerns with public attitudes (7)	7–28	160	18.5 (5.2)	2.1/3.1	0.877

Negative correlations were found between all subscales of the HIV stigma scale and the emotional well-being scales of Swed-Qual (Table 5). The correlation coefficients were of moderate size for the dimensions of *personalised stigma, negative self-image* and *concerns with public attitudes*. The magnitude of the correlation coefficients for the *disclosure concerns* dimension were small, however both exceeding 0.20.

**Table 5:** Bivariate correlations^a^ of the HIV stigma scale subscales and selected Swed-Qual subscales.

	Swed-Qual	Swed-Qual
HIV stigma scale	Emotional well-being: positive effect	Emotional well-being: negative effect
Personalised stigma	−0.297***	−0.397***
Disclosure concerns	−0.237**	−0.210**
Negative self-image	−0.426***	−0.494***
Concerns with public attitudes	−0.288***	−0.389***

a Spearman's rank correlation coefficient;

**p<0.01;

***p<0.001.

### Data Quality

The percentage of missing values for each item is presented in Table 3. The level of missing responses exceeded 5% for 18 of the items (5.2–10.8%). A majority of these items (n = 14) include an assumption that at least some people would know about the respondent's HIV status. Most of these items (n = 12) belonged to factor 1, *personalised stigma*. In the margins of the forms, some participants wrote “nobody knows” and either skipped items or marked the response alternative “completely disagree” for these items. Participants with missing values skipped either all the items for the *personalised stigma* dimension or skipped some items and chose the response alternative “completely disagree” on some. To secure that the instrument is valid and reliable for people without including this dimension we performed an exploratory factor analysis without the questions belonging to the dimension personalised stigma. Parallel analysis indicated a three factor solution with all items except item 37 loading on the same factors as in the four factor solution, as presented in Table 6.

**Table 6:** Factor loadings for items in the HIV stigma scale, items belonging to the dimension personalised stigma excluded.^a^

	Component		
Items	2	3	4
17. I am very careful who I tell that I have HIV	0.764		
6. I work hard to keep my HIV a secret	0.739		
1. In many areas of my life, no one knows that I have HIV	0.724		
21. I never feel the need to hide the fact that I have HIV (R)	0.617	0.327	
4. Telling someone I have HIV is risky	0.612		
22. I worry that people may judge me when they learn I have HIV	0.470	0.324	
25. I worry that people who know I have HIV will tell others	0.421		
37. I have told people close to me to keep the fact that I have HIV a secret		0.325	
15. Having HIV makes me feel that I'm a bad person		0.787	
7. I feel I am not as good a person as others because I have HIV		0.762	
3. People's attitudes about HIV make me feel worse about myself		0.666	
23. Having HIV in my body is disgusting to me		0.656	
12. Having HIV makes me feel unclean		0.649	
2. I feel guilty because I have HIV		0.635	
8. I never feel ashamed of having HIV (R)		0.566	
13. Since learning I have HIV, I feel set apart and isolated from the rest of the world		0.504	−0.361
20. Most people are uncomfortable around someone with HIV			−0.840
9. People with HIV are treated like outcasts			−0.657
14. Most people think that a person with HIV is disgusting		0.358	−0.605
10. Most people believe that a person who has HIV is dirty			−0.589
5. People with HIV lose their jobs when their employers find out			−0.578
16. Most people with HIV are rejected when others find out			−0.488
19. Since learning I have HIV, I worry about people discriminating against me		0.362	−0.511
11. It is easier to avoid new friendships than worry about telling someone that I have HIV			−0.360

a Factor loadings based on alpha factoring with oblimin rotation. Pattern matrix. Factor loadings <0.32 not shown.

(R) indicates that the item is reversely scored.

All response alternatives in the four-point Likert scale were used for all items. Floor effects ranged between 0.5 and 7.2% and ceiling effects between 1.5 and 9.3% (Table 4).

### Reliability

Cronbach's α for the subscales ranged from 0.871 to 0.958 (Table 4), and the total scale including all 39 items generated an α of 0.958.

## Discussion

In this study, the HIV stigma scale has been evaluated in a Swedish context with regard to psychometric properties. After excluding one item due to low factor loadings, the instrument, measuring four dimensions of stigma, was shown to have satisfactory construct validity and reliability. The instrument generated data of good quality, with the exception of items that assume that the participant has disclosed her or his HIV status to another person. The exploratory factor analysis supported the notion that the HIV stigma scale measures four dimensions of stigma in a Swedish context, as previously presented by Berger et al [15] in an American context; the content of the factors were interpreted to well represent the dimensions of *personalised stigma, disclosure concerns, negative self-image* and *concerns with public attitudes*. One item (item 11), *It is easier to avoid new friendships than to worry about telling someone I have HIV,* had no factor loadings >0.32, indicating that the item had a weak connection to all four stigma dimensions. Based on this, we recommend the item to be excluded when the HIV stigma scale is used in Sweden.

When the original English version of the HIV stigma scale was developed, 16 items cross-loaded with high loadings on several factors. When designing the subscales, Berger et al [15] suggested that these items be assigned to several subscales and that subscale scores should be computed by summing the responses of all items belonging to each factor. In the factor structure performed in the current study, only five items cross-loaded. To avoid an item's appearance in more than one subscale, we recommend assigning each item to the one factor that it had highest correlation with. The summation of item scores into subscale scores is justified if the item scores are roughly equivalent across the subscale and the corrected item-total correlation exceeds 0.4 for all items [31], which is supported by our analysis on a subscale level. We therefore recommend that subscale scores be computed by summing the responses for the items belonging to each subscale. In the original HIV stigma scale, it is also suggested that all item scores be summed into a total stigma score. Since the number of items varies across the subscales and the item means differed between subscales, the use of a total score can be questioned.

The correlation coefficients between the HIV stigma scale and measures of emotional well-being indicated that people reporting more stigma experienced fewer positive emotions and more negative emotions. The moderate correlation coefficient between the *personalised stigma*, *negative self-image* and *concerns with public attitudes* subscales and emotional well-being supports convergent validity for these subscales. The correlations for the *disclosure concerns* subscale were also in the expected negative direction but of small magnitude indicating a somewhat weaker relationship than the other three dimensions.

All four response alternatives were used for all items, which provide evidence that a four-point Likert scale is sufficient. Our analysis of floor and ceiling effects met the standards [34] for all subscales, which indicates that the scale measures an accurate depth of the concept.

We found a high rate of missing answers among items that assume at least some extent of disclosure of the diagnosis, mostly belonging to the dimension of *personalised stigma*. In many of the cases with missing answers in the dimension of *personalised stigma*, the participant had skipped several of the items for this particular dimension. Based on written responses in the margins of the questionnaires, we conclude that these participants had not disclosed their HIV status to anyone and were thus not able or did not find it meaningful to imagine the situations as requested in the instructions for the items assuming that the participant's HIV status is known to other people. We suggest that the instructions requesting the participant to imagine the situation should be removed since some participants skipped these items rather than gave an answer based on imagination. We instead recommend that the instrument include a fifth response alternative, “not applicable”. We recommend an additional item where the participant can state how many persons that, except healthcare providers, know about their HIV infection. If no-one except healthcare providers knows about the participant's HIV infection, they should skip all questions belonging to the dimension personalised stigma. The three-factor solution presented in Table 6 indicates that the instrument has construct validity even when the dimension personalised stigma is excluded. Item 37 have low loadings in this solution but we recommend keeping it in the dimension disclosure concerns due to its theoretical suitability for this dimension.

The item focusing on the risk of losing employment due to having HIV (item 5) had the highest non-response rate and may reflect the strong legal protection against this type of discrimination in Sweden. Based on written responses in the form (‘Does this happen in Sweden?’), it can be assumed that some participants found this item irrelevant and therefore did not answer. However, 38% of the participants agreed or totally agreed, which indicates relevance for some participants. Item 9, *People with HIV are treated like outcasts*, had a rate of missing answers that exceeded the limit set at 5%. This statement can evoke negative reactions among participants, but so can the subject of stigma as a whole. We do not see the missing values for these items as reason to exclude the items from the scale, but we suggest that someone be available to answer questions when the instrument is distributed. The HIV stigma scale is not recommended to be distributed by mail.

### Methodological Considerations

When evaluating exploratory factor analysis MacCallum et al [35] argue that the structure of the model and the communalities are of greater importance than the ratio of sample size to number of items. Further, in their Montecarlo study, MacCallum et al [35] shows that exploratory factor analysis can yield reliable solutions for sample sizes below 100 when communalities are high (overall >0.6) or wide (ranging from 0.2 to 0.8) and factors are over-determined (simple structure and high loadings on at least 3–4 items per factor) [35]. Our sample with 132 complete ratings for the full HIV stigma scale generates a solution with over-determined factors and wide communalities ranging from 0.35 to 0.80, why we believe that it is possible to draw firm conclusions based on the sample.

Studies that use patient-reported data from people living with HIV generally face a problem of representativeness. Many studies, including the original paper about the HIV stigma scale [15], had an overrepresentation of MSM and an underrepresentation of people of younger age, people with intravenous drug use and people from ethnic minorities [36]. The representativeness of the sample in this paper when compared to people living with HIV in Sweden [2] was evaluated using the χ^2^ goodness of fit test, showing that no significant difference could be found regarding gender and origin. The slight overrepresentation of participants with heterosexual path of transmission reflects the flow of patients at the clinic where the data collection was conducted.

## Conclusions

The HIV stigma scale, reduced by one item to 39 items, was shown to be a valid and reliable measure of four dimensions of stigma in a Swedish context. Response alternatives are suggested to be slightly revised for items assuming the disclosure of diagnosis to another person. We recommend that the items belonging to the personalised stigma dimension should be skipped for people that have not disclosed their HIV infection to anyone except their healthcare provider. Our analysis confirmed construct validity of the instrument even without this dimension.
